# Determining optimal cadence for an individual road cyclist from field data

**DOI:** 10.1080/17461391.2016.1146336

**Published:** 2016-02-22

**Authors:** Robert Reed, Philip Scarf, Simon Adrian Jobson, Louis Passfield

**Affiliations:** ^a^ Centre for Sports Business, Salford Business School, University of Salford, SalfordM5 4WT, UK; ^b^ Department of Sports Studies, University of Winchester, Sparkford Road, WinchesterSO22 4NR, UK; ^c^ School of Sport and Exercise Sciences, University of Kent, Medway Building, Chatham, KentME4 4AG, UK

**Keywords:** Cycling, power, heart-rate, training load, TRIMP

## Abstract

The cadence that maximises power output developed at the crank by an individual cyclist is conventionally determined using a laboratory test. The purpose of this study was two-fold: (i) to show that such a cadence, which we call the optimal cadence, can be determined using power output, heart-rate, and cadence measured in the field and (ii) to describe methodology to do so. For an individual cyclist's sessions, power output is related to cadence and the elicited heart-rate using a non-linear regression model. Optimal cadences are found for two riders (83 and 70 revolutions per minute, respectively); these cadences are similar to the riders’ preferred cadences (82–92 rpm and 65–75 rpm). Power output reduces by approximately 6% for cadences 20 rpm above or below optimum. Our methodology can be used by a rider to determine an optimal cadence without laboratory testing intervention: the rider will need to collect power output, heart-rate, and cadence measurements from training and racing sessions over an extended period (>6 months); ride at a range of cadences within those sessions; and calculate his/her optimal cadence using the methodology described or a software tool that implements it.

## Introduction

Many laboratory-based studies have sought to determine an optimal cadence (Coast & Welch, 1985, 90–105 rpm; Eckermann & Millahn, 1967, 30–60 rpm; Hagberg, Mullin, Giese, & Spitznagel, 1981, 80–90 rpm; Wildrick, Freedson, & Hamill, 1992, 35–57 rpm). Little consensus has emerged from these studies with some arguing that high cadences (preferred by professional cyclists) are optimal (Hagberg et al., 1981), that optimal cadence varies with work-rate (Foss & Hallen, 2004), or that high cadences are not optimal (Jacobs, Berg, Slivka, & Noble, 2013; Stebbins, Moore, & Casazza, 2014). There is some agreement that very high cadences are inefficient. This is likely due to factors such as the cost of moving the lower limbs (Winter & Knudsen, 2011), the muscle fibres involved exceeding their most efficient contractile velocities, and the increase in energy needed to stabilise the upper body (Hagberg et al., 1981; Leirdal & Ettema, 2011; Samozino, Horvais, & Hintzy, 2006). However, most of the above studies are concerned with identifying the optimal cadence in terms of cycling efficiency. Abbiss, Peiffer, and Laursen (2009) point out that the ideal cycling cadence may differ according to criteria adopted. They then state that the cadence chosen by cyclists may be selected according to whether it is the most economical, produces higher power output or reduces fatigue, or simply feels more comfortable. Ultimately, they conclude that the optimal cadence and criteria for its choice for an individual cycling task remain unclear.

Given the limitations of general conclusions about optimal cadence, a different field-based approach may be useful. With the advent of portable cycling power meters it may be possible to determine an optimal cadence for individual road cyclists from data gathered in the field using measures of their power output, cadence, and heart-rate. This change to the typical scientific paradigm presumes that analysis of cyclists’ historical field data may provide an insight into the cadences that cyclists select and the correlates that are significant in this choice. To seek optima from a range of uncontrolled field observations rather than from laboratory-controlled manipulations raises some key issues regarding: the practicality of obtaining a meaningful estimate of optimal cadence from field data; the appropriate methodology to obtain such an estimate; and the limitations of this methodology. To address these issues this study defines optimal cadence as that which maximises power output developed for a given heart-rate. For practical purposes this definition of optimal cadence is adopted because heart-rate can be interpreted as a proxy for metabolic work-rate (Churchill, Sharma, & Balachandran, 2009). Further, power output developed at the crank and heart-rate are easily measured in the field; and the definition allows the cadence that maximises power output for a given heart-rate to vary with heart-rate, paralleling the idea that the cadence that maximises gross efficiency may vary with the work-rate (Chavarren & Calbet, 1999; Coast & Welch, 1985; MacIntosh, Neptune, & Horton, 2000; Seabury, Adams, & Ramey, 1977). Finally, to our knowledge this study presents the first attempt to calculate an optimal cadence from field data on power output, heart-rate, and cadence. The most closely related work is that of Sassi, Rampinini, Martin, and Morelli (2008) who used field data to relate cadence, freely chosen by the riders, to road gradient, but without taking account of heart-rate.

## Methods

### Description of the participating riders and their field data

**Figure 1.**
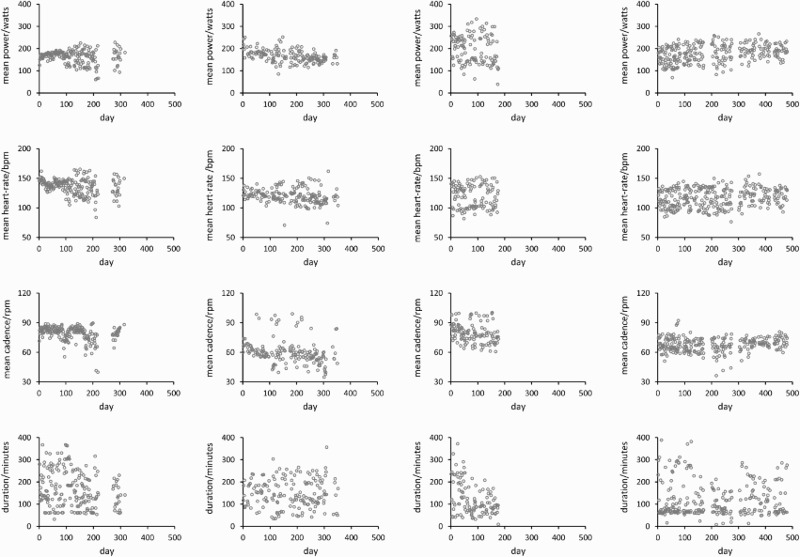
Means for each training session for each rider. Rows correspond to power output (watts), heart-rate (bpm), cadence (rpm), and duration (minutes); columns corresponding to riders 1–4.

The methodology developed is illustrated using the cycling data of four competitive, male riders. These riders collected data on power output, heart-rate, and cadence for nominally all their sessions over a period in 2006–2008. Unlike in the laboratory tests mentioned above, there was no prescription regarding cadence control and cyclists rode with wide-ranging cadence, heart-rate, and power output. At the time the data were collected the ages, masses, and heights of the riders were 21, 40, 52, 45 years, 61, 76, 75, 74 kg, and 171, 178, 175, 183 cm. Power output was measured using power-meter cranks (Schoberer Rad Messtechnik (SRM), Julich, Germany); the sampling interval for the three variables was 5 seconds. Although the data were not collected specifically for this study, the riders gave written, informed consent for their data to be used in this study, and the study received ethics committee approval at the University of Kent and was carried out according to the principles of the Declaration of Helsinki (World Medical Association, 2013). The cranks were all serviced and calibrated by SRM immediately prior to the data collection period. The calibration of the cranks was verified part way through the data collection period using calibrated weights (Wooles, Robinson, & Keen, 2005). The mean power output, mean heart-rate, mean cadence, and duration of each recorded session for the four riders are shown in Figure 1. The authors did not know: (a) if a session was racing or training and (b) if a rider collected data for every one of their sessions over the period of data collection. This is only problematic if the relationship between power output, heart-rate, and cadence differs between training and racing or if some factor related to these variables has influenced the presence/absence of data; this is unlikely.

### Power output and heart-rate relationship

In the empirical models described below, power output is related to heart-rate and cadence. The nature of the power output, heart-rate relationship, and the time-lag between the power output and the heart-rate response must be specified. Grazzi et al. (1999) conclude from a study with 500 tests and 290 participants that the relationship is to a large extent proportional (correlation 0.98 for heart-rates between 90 bpm and 180 bpm). The lag between a change in power output and the heart-rate response is less clear from literature. Jeukendrup and van Diemen (1998) discuss the basis of the lag, but do not indicate its size. Stirling, Zakynthinaki, Refoyo, and Sampredo (2008) conclude, from a study in which one 33-year-old male undertook a track running session with five efforts and 10 minutes rest between each effort, that large changes (up and down) in heart-rate occur over 30–60 seconds. For smaller changes, the time lag may be considerably less. Consequently, this paper experiments with a heart-rate lag, denoted by *l*, of between 5 and 60 seconds*.*

### Data processing

To reduce serial dependence, the 5-second observations were systematically sampled every *m*th measurement. The explanatory power of the models is not sensitive to *m*, and so the sampling interval is set at 120 seconds (*m* = 24). Sessions were combined to provide one large series for an individual rider. Alternatively, a session effect might be modelled as a random effect in a mixed model, but this is not pursued further. Instead this study uses other within-session and between-sessions explanatory variables to represent session and long-term training load effects. Outliers that were the result of miss-recording were removed. Finally, only those recorded measurements for which heart-rate was higher than the individual's mean heart-rate were used in the model fitting, since such data will be more representative of competition than the complete record.

### An empirical model of power output, heart-rate, and cadence

While heart-rate principally acts as a response to power output, cadence, and other training related variables, it is convenient, for determining optimal cadence, to invert this relationship and to regard power output as explained by the covariates. The model we propose for an individual rider is
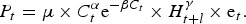



where 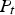 is the power output at time *t*, 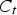 is the cadence at time *t*, 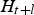 is the heart-rate (response) *l* time units later (within the same session), 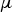 is a constant, and the error term is such that 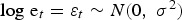 (independent). The parameters 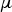, *α*, *β*, 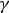, and 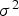 must be estimated from the field data. This model has the desirable properties: (a) power output is zero when cadence is zero; (b) when 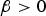 there exists a cadence at which the mean power output is maximum. The simpler quadratic model 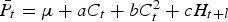 possesses the property (b) (when *b < *0) but not the property (a). Although the response variable in this regression model is power output, the model does not imply that power output is caused by the heart-rate but merely that power output is related to the heart-rate at some time in the future.

The logarithmic transform(1) 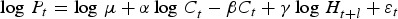



was fitted by ordinary least squares using the R software package (R Development Core Team, 2005). Equation (1) implies that the mean power output at time *t* is(2) 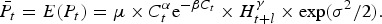



Model (1) might be modified so that the power output is related to the heart-rate excess, 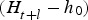, where 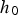 is the resting heart-rate: 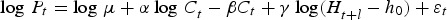. Then the model possesses the further desirable property that when 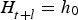, 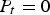. This model was fitted but did not change the estimates of the key cadence parameters, *α* and *β*, because, perhaps, only those observations with “above average” heart-rate were used.

The model (1) might also be refined to account for serial dependence. However, least squares estimation of covariate effects is robust to serial dependence, although the standard errors of estimates are underestimated. Furthermore, the linear regression model with first-order autocorrelated errors (Kariya & Kurata, 2004, p. 25) assumes that covariates themselves are not autocorrelated, which is not the case here. Therefore, it is sensible to use systematic sampling.

### Optimal cadence

The optimal cadence, according to the definition in this study, is obtained by differentiating equation (2) with respect to cadence, regarding the other variables as constants, equating the result to zero, and solving for the cadence. It is more convenient to differentiate the logarithm of the expected power output (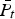 will be maximised when log 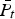 is maximised). The subscript *t* is dropped since it is implied that the expected power output developed and cadence applied are concurrent. Thus, 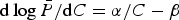, so that 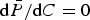 when 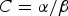. This cadence maximises the expected power output, provided the second derivative is negative, that is, if 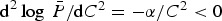, and hence if 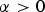, and is also positive if 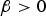. Thus, if 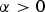 and 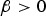,(3) 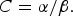



is the optimal finite, positive cadence and is denoted by 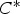. The heart-rate coefficient *γ* should be positive and near 1 since power output and heart-rate are broadly proportional (Grazzi et al., 1999).

The estimated optimal cadence is found by substituting model parameter estimates into (3). Confidence intervals for optimal cadence can be found using the delta method (Casella & Berger, 2002, p. 240): the variance of 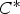 is approximately 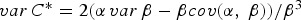, so that an approximate 95% confidence interval is 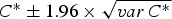.

To describe the practical significance of an optimal cadence (if it exists), it is informative to use the interval of cadence over which the expected power output varies by at most *r* percent below the expected power output at the optimal cadence.

### Training load covariates

To account for the possible effect of the accumulation of fatigue on the power output, heart-rate, and cadence relationship and hence on the optimal cadence, two measures of training load related to the training impulse are calculated.

The training load accruing from a particular session at time 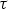 within the session is defined as(4) 
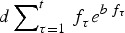



where 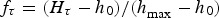 is the heart-rate fraction reserve at time 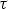 within a session, 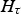 is the heart-rate at time 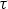, and 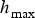 and 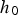 are the maximum and resting heart-rates for the individual rider. This quantifies the within-session training load dynamically. In the manner of the classic definition of TRIMP (Morton, Fitz-Clarke, & Banister, 1990), 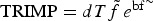), it puts more weight on instances with a high heart-rate when heart-rate varies within a session. As in Morton et al. (1990), *b* is set to 1.92. Resting heart-rate was self-reported and taken in the morning upon waking; maximum heart-rate was the highest recorded in the participant's data. TRIMP is a dimensionless quantity, although it is important that the time units used in the calculation of 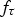 and *d* are consistent; that is, if heart-rate is measured in beats per minute then duration should be measured in minutes, so that the classic TRIMP measure (heart-rate × duration, *dT*) is the total number of heart beats in a session (Jobson, Passfield, Atkinson, Barton, & Scarf, 2009). When this new TRIMP term (expression 4) interacts with cadence in the regression model, variation in optimal cadence within a session can be explored. That is, the model will allow the possibility that optimal cadence changes as a rider tires during a session.

To quantify the cumulative effect of training loads of previous sessions on the current session, a cumulative TRIMP is defined as follows. The session TRIMP (expression 4 with *t* set to the final time point of the session) is calculated. For session *i* denote this by *X_i_* . Then in the spirit of the Banister model (e.g. Calvert, Banister, Savage, & Bach, 1976), the cumulative TRIMP at session *i* is a weighted sum of previous session TRIMPs such that more weight is given to more recent sessions: 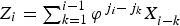. where 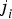 is the day number of session *i* (taking the first day of the training schedule as day 1). The decay coefficient 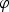 determines how much weight is given to the most recent sessions. This cumulative TRIMP is then introduced into the regression model in two possible ways:(5) 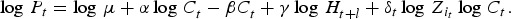

(6) 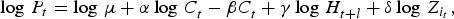



where 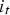 is the session number at time *t.* Here cadence is either interacting with TRIMP (equation 5) or not (equation 6). With the interaction term present, the optimal cadence is 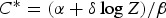, so that the optimal cadence is training load (TRIMP) specific. The within-session TRIMP is introduced into the model in a similar way, so that an interaction term allows the optimal cadence to vary within a session, according to the training load (fatigue) experienced within the session so far. In both approaches, the TRIMP term in the model allows power output to vary with training load, thus modelling the effect of within-session (short-term) fatigue or accumulated (long-term) fatigue. It may also be interesting to consider whether optimal cadence varies over multiple sessions as a consequence of changing fitness. This effect is discussed in passing by Passfield and Doust (2000). However, a study of such an effect is beyond the scope of this paper.

The criterion used for selecting the best model is minimum AIC (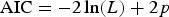, where *L* is the log-likelihood value and *p* the number of parameters (Kendall, Stuart, Ord, & Arnold, 1999, p. 748)). The explanatory power (*R* ^2^), the amount of variation in the response variable explained by the explanatory variables, is also used to compare models with the same number of parameters, and for measuring the effectiveness of additional covariates. Finally, the values of parameter estimates themselves can be used since 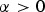 and 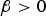 is required for a finite, positive optimal cadence to exist.

## Results

**Figure 2.**
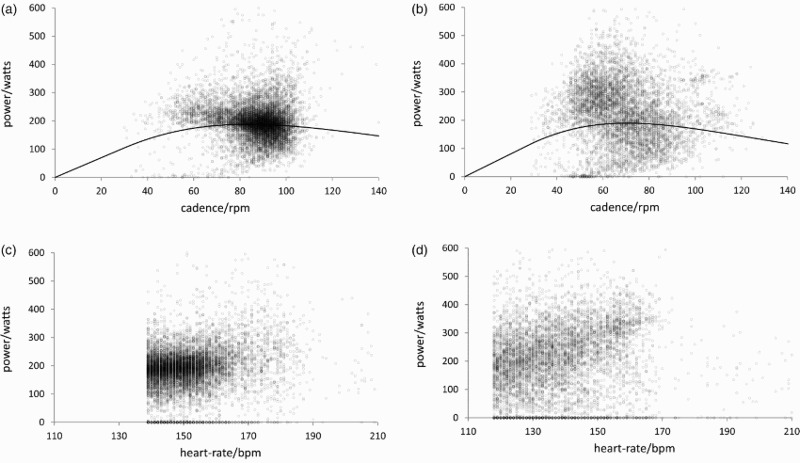
Power output (watts) vs. cadence (rpm) and heart-rate: (a,b) rider 1; (c,d) rider 2. Solid line: fitted, expected power output from the model equation (1) with heart-rate 151 bpm (rider 1), 139 bpm (rider 2); heart-rate lag 30 seconds.

Table I shows the results for the basic model (equation 1) for heart-rate lags, *l,* of 1, 2, 6, and 12 time units. The positive values of *α* and *β* for riders 1 and 2 thus yield (from equation 3) optimal cadences of 83 ± 1 rpm and 70 ± 1 rpm, respectively. Negative *β* for riders 3 and 4 imply that optimal cadences cannot be determined. Indeed the model is a better fit for riders 1 and 2 than for riders 3 and 4 in many respects: standard errors of coefficients are smaller; explanatory power (*R* ^2^) is higher. The explanatory power of the model is greatest for *l* *=* 6 (30 seconds). Table II considers the practical significance of the estimated optimal cadences. The riders preferred cadences were 82–92 rpm and 65–75 rpm, respectively. Figure 2 shows the power output against cadence for riders 1 and 2, and the fitted power-outputs. The preferred cadences are inferred directly from these data. Figure 2 also shows the power output against heart-rate, noting that we have discarded data in which the heart-rate is less than the mean, rider-specific heart-rate for that rider.

**Table I.** Coefficient estimates (standard errors in parenthesis) and explanatory power for the power output/heart-rate model, equation (1), for each rider and for different values of heart-rate lag, *l.*

	*l*	*α*	*β*	*γ*	log μ	*R*^2^	AIC
Rider 1	1	1.58 (0.01)	0.021 (0.0005)	0.91 (0.06)	−4.59 (0.29)	84.0	69,664
	2	1.56 (0.01)	0.020 (0.0005)	0.90 (0.06)	−4.51 (0.31)	84.2	70,919
	6	1.53 (0.01)	0.018 (0.0006)	0.77 (0.07)	−3.88 (0.33)	85.9	72,600
	12	1.51 (0.01)	0.017 (0.0006)	0.71 (0.07)	−3.55 (0.35)	85.8	72,727
Rider 2	1	1.66 (0.02)	0.024 (0.0008)	0.75 (0.08)	−3.71 (0.42)	74.7	50,095
	2	1.65 (0.02)	0.024 (0.0009)	0.84 (0.09)	−4.15 (0.44)	75.9	50,659
	6	1.61 (0.02)	0.023 (0.0010)	0.61 (0.10)	−2.99 (0.49)	76.5	51,362
	12	1.56 (0.02)	0.020 (0.0010)	0.28 (0.11)	−1.36 (0.51)	75.7	52,218
Rider 3	1	1.17 (0.03)	−0.0015 (0.001)	1.06 (0.14)	−5.19 (0.68)	58.9	32,595
	2	1.13 (0.03)	−0.0033 (0.001)	1.05 (0.14)	−5.14 (0.70)	61.2	33,024
	6	0.99 (0.03)	−0.0095 (0.001)	1.23 (0.15)	−5.95 (0.75)	63.5	33,228
	12	0.95 (0.04)	−0.0113 (0.002)	0.92 (0.15)	−4.45 (0.75)	63.6	33,156
Rider 4	1	0.15 (0.03)	−0.0007 (0.002)	1.47 (0.14)	−2.59 (0.69)	8.7	82,794
	2	0.09 (0.03)	−0.0055 (0.002)	0.09 (0.15)	−4.08 (0.77)	9.2	83,476
	6	0.18 (0.04)	−0.0020 (0.002)	1.47 (0.16)	−2.84 (0.78)	11.1	84,747
	12	0.20 (0.04)	−0.0012 (0.002)	1.72 (0.17)	−4.25 (0.82)	12.6	85,994

**Table II:** Fitted, expected power output for model (equation 1) for a range of cadences above and below the statistically optimum cadence, along with the percentage reduction in power output for each sub-optimal cadence. Rider 1 (left) and rider 2 (right), for heart-rate lags of 30 seconds, at heart-rates of 151 and 139 beats per minute for riders 1 and 2 respectively.

Change in *C* from C*	Cadence	Expected power output	Change in power output	% change in power output	Change in *C* from C*	Cadence	Expected power output	Change in power output	% change in power output
−20	63.4	178.2	−9.6	−5.1	−20	50.4	174.9	−14.7	−7.7
−10	73.4	185.5	−2.2	−1.2	−10	60.4	186.2	−3.4	−1.8
−5	78.4	187.2	−0.5	−0.3	−5	65.4	188.8	−0.8	−0.4
0	83.4	187.8			0	70.4	189.6		
5	88.4	187.3	−0.5	−0.3	5	75.4	188.8	−0.7	−0.4
10	93.4	185.9	−1.9	−1.0	10	80.4	186.8	−2.8	−1.5
20	103.4	180.8	−7.0	−3.7	20	90.4	179.5	−10.1	−5.3

Introducing terms in the model corresponding to the accumulated fatigue (models 5 and 6) did not yield significant improvements *R* ^2^. Results were likewise when the within-session TRIMP variable was considered. Also, an additional term in the model for the interaction between heart-rate and cadence was non-significant, implying that an optimal cadence if it exists does not depend on the heart-rate and hence the level of power output. Full details of the results of these particular analyses are given in Reed (2013).

## Discussion

The variability in the power output–cadence relationship (Figure 2) is very large. However, heart-rate variation explains much of this variability for riders 1 and 2 (*R* ^2 ^= 84 and 75%, respectively). This is not the case for riders 3 and 4 (*R* ^2 ^= 59 and 9%, respectively). It is not surprising therefore that an optimal cadence for rider 4 cannot be estimated from these data, and it is reasonable to suppose that satisfactory estimation of an optimal cadence requires a good power output-cadence “signal”. Thus, successful determination of an optimal cadence may depend as much on the variability in the riding of the rider as on the biomechanics/physiology of the rider. If a rider does not vary his/her cadence much, then there will be scant information about the heart-rate/power output/cadence relationship in their field data record. Indeed riders 1 and 2 in our study appeared to train at a variety of cadences, while the cadence of rider 4 was less variable. Rider 3 had a shorter training record.

In the methodology, heart-rate lag is a critical parameter. The literature suggests a value of 30 seconds for this parameter and the statistical evidence from the model fitting provided further support for this value.

The estimated optimal cadences obtained for riders 1 and 2 are both statistically significant (on the basis of the sizes of the standard errors of the cadence coefficients *α* and *β* (Table I) in the model) and practically significant (on the basis of the reduction in power output when cadence is sub-optimal, Table II ). Riding at a cadence 20 rpm below optimum yields a mean power output reduction of 9.6 watts (5.1%) for rider 1 and 14.7 watts (7.7%) for rider 2. The optimal cadences tally with the riders’ preferred cadences.

A statistically significant effect does not establish a cause. An underlying, instrumental variable may be the cause (see e.g. Angrist, Imbens, & Rubin, 1996). For example, for a given heart-rate, preferred cadences and power output may be changed by the training load and this may be reflected in a higher cardiovascular drift (Jeukendrup & van Diemen, 1998; Wingo, Lafrenz, Ganio, Edwards, & Cureton, 2005). To control for this potential effect, variables that quantify short-term and long-term training load are fitted, but do not have statistically significant effects. This does not mean that there is no effect on optimal cadence: if there is a linear effect, this analysis failed to detect it. The actual relationship between expected power output and within-session TRIMP may in fact be non-linear: there may be both a warm-up effect and a progressive increase in heart-rate response during a session. Physiological systems will be fully functioning only after some time, so that, up to a point, a cyclist becomes increasingly efficient as more TRIMPS are banked. Furthermore, additional factors that will influence heart-rate response to a given power output are ambient temperature, altitude/barometric pressure (unlikely to have changed notably in this study), and dehydration. Unless a cyclist completely replaces fluids lost through sweating, their heart-rate response should increase progressively during exercise. On a hot day and a long ride this change could exceed 10 bpm from beginning to end. The relationship between power output and within-session TRIMP may therefore be an inverted “U” of some kind. It would be a significant challenge to model the within-session TRIMP in this way, although given data relating to such additional factors an extended study could be envisaged. Also, long-term fatigue may be compounded with increasing fitness. Gradient may also affect the optimal cadence for a given power output (Arkesteijn, Jobson, Hopker, & Passfield, 2013), so that hilly and flat training rides may make a difference to the optimal cadence.

This study does not measure the intensities of training sessions, and no direct information (e.g. via training diaries) about the nature of specific sessions was available. Models with other covariates were fitted to the data but no conclusive results were obtained. Such covariates related to: cumulative short-term session duration; session variables calculated using the concept of normalised power output; and interactions terms. In particular, a heart-rate/cadence interaction term, which allows the possibility of optimal cadence varying with heart-rate, did not improve the model fit, so that for these riders optimal cadence does not appear to be power output dependent. Finally, heart-rate lag (*l*) is a key model parameter that must be chosen carefully. In this study, for riders 1 and 2 the appropriate value (based on the explanatory power of the model) was 30 seconds (*l* *=* 6). This value concurs with other studies in the literature (e.g. Stirling et al., 2008).

Riders 1 and 2 were the younger riders in this study, although no conclusion should be drawn from this. Consideration of an age effect would require further investigation. Indeed we reiterate that this study does not seek to draw conclusions on general physiological or biomechanical mechanisms. We merely present a methodology for determining for an individual rider the cadence that maximises his/her power output for a given heart-rate.

## Conclusions

This study presents methodology for estimating optimal cadence for individual road cyclists from data on within-session power output, heart-rate, and cadence. The methodology supposes that power output is explained by rider specific heart-rate and cadence, and uses non-linear regression to model the relationship between these variables. Optimal cadences are yielded for two of the four riders in the study (83 and 70 rpm, respectively). These values concur with the riders’ own preferred cadences (82–92 rpm and 65–75 rpm, respectively). The optimal cadences are practically significant because variation from optimal cadence appears to lead to important reductions in power output.

The study of the effects of short- and long-term training load, through the inclusion in the model of covariates that measure training load, was inconclusive. The fitted model did not consider a session effect. Instead all data were combined into one stream for each rider. Ideally, the nature of a session recorded through a training diary would be available and could be used as a covariate. The study does however focus on higher intensity power output by only considering those parts of sessions for which the heart-rate was above average.

The proposed methodology could be implemented by any road cyclists to calculate their indivdual optimal cadence: a cyclist will need to collect power output, heart-rate, and cadence measurements from training sessions over an extended period (>6 months); ride at a range of cadences within those sessions; and calculate his/her optimal cadence periodically to take account of possible changes in fitness. While the participants were not elite cyclists, this does not a limit the study as the methodology is necessarily rider specific. The heart-rate lag is an important parameter, and this needs to be chosen carefully.

An important limitation is that the fitted model does not account for whether the cyclist is riding in or out of the saddle. Optimal cadence may be specific not only to a rider but also to his/her mode of riding. In principle, the methodology can accommodate riding mode if it is measured, either through a rider controlling and reporting riding mode or through some monitoring device located on the saddle.

*Twitter promotion:* methodology to find a rider's best cadence using power, heart-rate, and cadence collected on the bike
